# Whole genome sequencing of *Salmonella* Chester reveals geographically distinct clusters, Norway, 2000 to 2016

**DOI:** 10.2807/1560-7917.ES.2019.24.4.1800186

**Published:** 2019-01-24

**Authors:** Lotta Siira, Umaer Naseer, Kristian Alfsnes, Nils Olav Hermansen, Heidi Lange, Lin T Brandal

**Affiliations:** 1 Division for Infection Control and Environmental Health, Norwegian Institute of Public Health (NIPH), Oslo, Norway; 2 European Program for Public Health Microbiology Training (EUPHEM), European Centre for Disease Prevention and Control (ECDC), Stockholm, Sweden; 3 Department of Microbiology, Oslo University Hospital, Oslo, Norway

**Keywords:** Salmonella, Salmonella Chester, laboratory surveillance, typing, whole genome sequencing, WGS, bacterial genomes, outbreak detection, Norway, food-borne infections, bacterial infections, salmonellosis, molecular methods, typing

## Abstract

**Introduction:**

During summer 2016, Norway observed an increase in *Salmonella enterica* subsp. *enterica* serovar Chester cases among travellers to Greece.

**Aim:**

Our aim was to investigate genetic relatedness of *S.* Chester for surveillance and outbreak detection by core genome multilocus sequence typing (cgMLST) and compare the results to genome mapping.

**Methods:**

We included *S.* Chester isolates from 51 cases of salmonellosis between 2000 and 2016. Paired-end sequencing (2 × 250 bp) was performed on Illumina MiSeq. Genetic relatedness by cgMLST for *Salmonella* *enterica* subsp. *enterica,* including 3,002 genes and seven housekeeping genes, was compared by reference genome mapping with CSI Phylogeny version 1.4 and conventional MLST.

**Results:**

Confirmed travel history was available for 80% of included cases, to Europe (n = 13), Asia (n = 12) and Africa (n = 16). Isolates were distributed into four phylogenetic clusters corresponding to geographical regions. Sequence type (ST) ST411 and a single-locus variant ST5260 (n = 17) were primarily acquired in southern Europe, ST1954 (n = 15) in Africa, ST343 (n = 11) and ST2063 (n = 8) primarily in Asia. Part of the European cluster was further divided into a Greek (n = 10) and a Cypriot (n = 4) cluster. All isolates in the African cluster displayed resistance to ≥ 1 class of antimicrobials, while resistance was rare in the other clusters.

**Conclusion:**

Whole genome sequencing of *S.* Chester in Norway showed four geographically distinct clusters, with a possible outbreak occurring during summer 2016 related to Greece. We recommend public health institutes to implement cgMLST-based real-time *Salmonella* *enterica* surveillance for early and accurate detection of future outbreaks and further development of cluster cut-offs.

## Introduction

Salmonellosis is characterised by gastroenteritis with acute onset of fever, abdominal pain, diarrhoea, nausea and occasionally vomiting and is one of the most commonly reported food-borne diseases in Europe. In 2016, 20 confirmed salmonellosis cases per 100,000 population were reported in the European Union (EU) [1]. During the summers of 2014 and 2015, several European countries reported an increase in cases with salmonellosis caused by *Salmonella enterica* subsp. *enterica* serovar Chester. This multi-country outbreak was associated with travel to Morocco and was probably linked to multiple food sources [2]. Previously, human cases of salmonellosis from *S.* Chester had rarely been reported, but after this outbreak, *S*. Chester was included among the 20 most common *Salmonella* serovars causing infections in humans in Europe in 2014 [3]. *S*. Chester has since accounted for 0.4% of the annually reported salmonellosis cases in Europe [1]. Outbreaks caused by *S*. Chester have also been reported elsewhere. In 2010, *S.* Chester was implicated in two outbreaks in North America: in Canada, head cheese (brawn) was identified as the source [4], and a multi-state outbreak in the United States (US) was associated with frozen meals [5]. In China, *S.* Chester was isolated in a multi-serovar *Salmonella* outbreak in 2012, where egg sandwiches were implicated as the main vehicle [6]. In Australia, *S*. Chester outbreaks associated with turtle meat and municipal water were described in 1998 and 2005, respectively [7,8].

**Figure 1:**
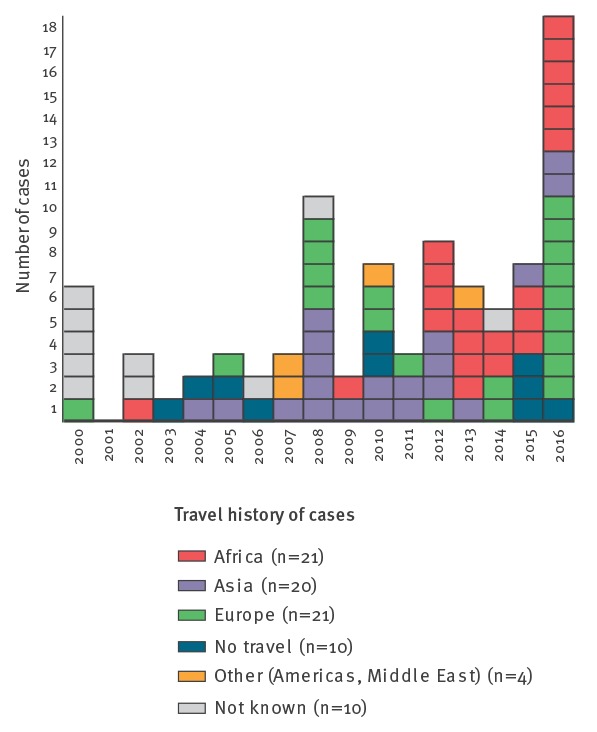
*Salmonella* Chester cases, by travel history and year, Norway, 2000–2016 (n = 86)

Salmonellosis has been notifiable to the Norwegian Surveillance System for Communicable Diseases (MSIS, http://www.msis.no/) since 1977, and the corresponding isolates are sent to the National Reference Laboratory for Enteropathogenic Bacteria at the Norwegian Institute of Public Health (NIPH). During the period from 2000 to 2016, the number of *S.* Chester cases by year in Norway has ranged between zero and 18. A travel history was confirmed in 78% of the cases; Europe, Asia and Africa were approximately equally represented as travel destinations of the cases (Figure 1). During summer 2016, we observed an increase in cases with a history of travel to the island of Rhodes, Greece. This information was shared with other European countries through the Epidemic Intelligence Information System (EPIS) of the European Centre for Disease Prevention and Control (ECDC), but the communication returned no reports of cases outside Norway.

In this study, we studied the genomic relatedness of *Salmonella* Chester isolates by whole genome sequencing (WGS) analysed by a core genome multilocus sequence typing (cgMLST) scheme and compared with results obtained by single nucleotide polymorphism (SNP)-based reference genome mapping. Our aim was to identify if the cases with a history of travel to Greece were part of the Moroccan outbreak cluster and to examine the molecular epidemiology of isolates with different geographical origins. Comparisons of cgMLST- and SNP-based results are important as the use of WGS is increasingly used in public health. This requires information on the diversity of sequences within a species, serovar and previously defined genotypes, to determine appropriate cut-offs for clusters and outbreaks. Our study contributes to this body of knowledge. As Norwegians travel frequently both within Europe and outside the continent [9] and the majority of the *Salmonella* cases identified in Norway report a history of travel, our surveillance data are well positioned to give insight into the internationally circulating *S.* Chester strains.

## Methods

### Cases and isolates

Fifty-one of 86 non-duplicate *S.* Chester isolates from the national strain collection at the National Reference Laboratory for Enteropathogenic Bacteria at NIPH from the years 2000 to 2016 were included in the study. These consisted of all isolates from the years 2014 to 2016 (n = 30) and a selection of older isolates from the period 2000 to 2013 (n = 21) chosen so that they were representative of the travel history of all cases. Isolates from all cases reporting travel to Greece, Cyprus or Morocco in the years 2000 to 2016 were included.

### Epidemiological investigations

*S*. Chester cases with a history of travel to Greece during summer 2016 were interviewed to obtain more detailed information on travel destination, dates of stay, accommodation, travel agency and foods consumed at the destination.

### Serotyping

Serotypes were confirmed by agglutination tests with antisera (Sifin Diagnostics GmbH, Berlin, Germany and SSI, Statens Serum Institut, Hillerød, Denmark) according to the White–Kauffmann scheme [10]. The SeqSero online tool, version 1.0 (http://denglab.info/SeqSero) was used to identify the serotype from the raw sequence reads [11].

### Whole genome sequencing

DNA extraction was performed by MagNAPure 96 (Roche Molecular Systems Inc., Pleasanton, US). KAPA HyperPlus (Kapa Biosystems, Wilmington, US) was used for library preparation and Agencourt AMPure XP (Beckmann Coulter Life Sciences, Indianapolis, US) for removal of adaptor dimers. WGS was performed as paired-end (250 bp × 2) sequencing on the MiSeq (Illumina, Inc., San Diego, US) platform aiming for coverage of > 50×. Quality control of the raw reads was done through FastQC. The sequences were submitted to the European Nucleotide Archive (ENA) under the access number PRJEB30485.

### Multilocus sequence typing

Genotyping by the *Salmonella enterica* seven-gene MLST scheme was performed through EnteroBase in the SeqSphere^+^  software, version 4.0 (Ridom GmbH, Münster, Germany) based on the Achtman scheme.

### Core genome multilocus sequence typing

All analyses were performed using SeqSphere^+^. Briefly, raw sequence reads were trimmed until an average Phred base quality of ≥ 30 was reached in a window of 20 bases, and de novo assembly was performed using Velvet version 1.1.04 with default settings. We used the SeqSphere^+^ integrated cgMLST scheme developed by Alikhan et al. for EnteroBase (https://enterobase.warwick.ac.uk/) [12], with allele calling procedure with a minimum accepted BLAST identity of 80%, no BLASTp search, frame-shift detection turned on and independent SeqSphere^+^ allele numbering nomenclature. The allelic profiles of the isolates were visualised as a neighbour-joining tree using the parameter ‘pairwise ignoring missing values’.

### Single nucleotide polymorphism-based reference mapping analysis

CSI Phylogeny, version 1.4 (https://cge.cbs.dtu.dk/services/CSIPhylogeny/) was used to map sequences of our strains under investigation against *S.* Chester SRX992125 as a reference using the Burrows-Wheeler Aligner (BWA), call and filter SNPs through SAMtools using default parameters [13,14]. In MEGA6, the maximum likelihood method based on the Tamura-Nei model was used to infer phylogeny from the reference-based SNP calling, and a bootstrap consensus tree inferred from 1,000 replicates was also produced [15,16].

### Discriminatory power

Calculations comparing the discriminatory power of MLST, cgMLST and reference mapping methods were performed using Simpson's index of diversity [17].

### Antimicrobial susceptibility testing

All isolates were routinely tested for ampicillin, ciprofloxacin (CIP) and trimethoprim-sulfamethoxazole using the agar disk diffusion method. From 2016 onwards, quinolone resistance was inferred from pefloxacin resistance (n = 18). In addition, a selection of the isolates (n = 32) were screened for tetracycline (TET), chloramphenicol and nalidixic acid (NAL). Results were interpreted as sensitive (S), intermediate (I) or resistant (R) using the EUCAST clinical breakpoints, version 7.1 [18] when available, or based on epidemiological cut-off values of national zone distributions for CIP (S ≥ 33 mm and R < 30 mm), NAL (R < 16 mm) and TET (R < 17 mm) [19].

### Characterisation of antimicrobial resistance determinants, single nucleotide polymorphisms associated with resistance and plasmids

The online tools ResFinder version 3.0, PlasmidFinder version 1.3 and pMLST version 1.4 available at the Center for Genomic Epidemiology (http://www.genomicepidemiology.org/) were used, respectively, for sequence-based identification of acquired resistance genes, known mutations conferring resistance and plasmid-borne genes, using assembled genomes obtained through SPAdes Genome Assembler version 3.0 (Algorithmic Biology Laboratory, St. Petersburg University, St. Petersburg, Russia) [20,21]. For PlasmidFinder, the threshold for minimum identity was set at 95% and for coverage at 80%. For ResFinder, the threshold for minimum identity was set at 90% and for coverage at 60%.

## Results

### Description of cases

Nine cases had no travel history outside Norway, the remaining 42 cases had a history of travel to southern Europe (n = 13), Africa (n = 16) or Asia (n = 12). The travel history of one case was unknown. The median age of the cases was 44 years (range: 7–86 years) and 26 of the 51 cases were male.

Seven of eight cases with a history of travel to Greece in the summer of 2016 were interviewed upon giving a sample positive for *S.* Chester; however, the only common exposure that was revealed was staying on Rhodes (7/7) with the majority staying in the city of Rhodes (6/7).

### Serotypes

Conventional and sequence-predicted antigenic profiles were concordant, identifying the serotype Chester (4:e,h:e,n,x) for all isolates. 

### Multilocus sequence typing and core genome multilocus sequencing typing

The isolates represented five MLST sequence types (STs): ST1954 (n = 15), ST411 (n = 14), ST343 (n = 11), ST2063 (n = 8) as well as ST5260 (n = 3) which is a single-locus variant (SLV) of ST411 (Table).

**Table:** Characteristics of *Salmonella* Chester strains included in the study, Norway, 2000–2016 (n = 51)

WGS cluster^a^	ST	Travel history	Year	Resistance determinants identified through ResFinder	PlasmidFinder and pMLST result	Phenotypic antimicrobial susceptibility				
						BL	CHL	QNL	SXT	TET
Europe (n = 17)	ST411 (n = 14)	Greece (n = 8)	2016	*QnrB19*	IncI1, IncX3(pEC14), Col(pVC)	S	ND	S	S	ND
			2016	*QnrB19*	Col(pVC)	S	ND	S	S	ND
			2016	*QnrB19*	None	S	S	S	S	S
			2016	*aph(6)-Id*	Col(pVC)	S	S	I	S	S
			2016	None	Col(pVC)	S	S	I	S	S
			2016	None	None	S	ND	S	S	ND
			2016	None	None	S	ND	S	S	ND
			2016	None	None	S	ND	S	S	ND
		None (n = 3)	2006	None	None	S	S	S	S	S
			2015	*QnrB19*	Col(pVC)	S	ND	I	S	ND
			2016	None	Col(pVC)	S	ND	S	S	ND
		Southern Europe (n = 1)	2016	None	None	S	ND	S	S	ND
		Ivory Coast (n = 1)	2012	None	None	S	S	I	S	S
		Cyprus (n = 1)	2000	None	IncI2	S	S	S	S	S
	ST5260 (n = 3)	Cyprus (n = 3)	2010	None	None	S	S	S	S	S
			2014	None	Col(pVC)	S	ND	I	S	ND
			2014	*aph(6)-Id, strA, tet(A)*	IncFII, Col(pVC)	S	ND	I	S	ND
Africa (n = 15)	ST1954 (n = 15)	Morocco (n = 14)	2013	*aph(6)-Id, aph(3”)-Ib, QnrS1, floR, sul2, tet(A), dfrA14*	Col(pVC), IncN_ST7	S	R	R	R	R
			2013	*aph(6)-Id, aph(3”)-Ib, QnrS1, floR, sul2, tet(A), dfrA14*	Col(pVC), IncN_ST7	S	R	R	R	R
			2013	*aph(6)-Id, aph(3”)-Ib, QnrS1, floR, sul2, tet(A), dfrA14*	IncN_ST7	S	R	R	R	R
			2014	*aph(6)-Id, aph(3”)-Ib, QnrS1, floR, sul2, tet(A), dfrA14*	Col(pVC), IncN_ST7	S	R	R	R	R
			2014	*aph(6)-Id, aph(3”)-Ib, QnrS1, sul2, tet(A), dfrA14*	Col(pVC)	S	ND	R	R	ND
			2015	*aph(6)-Id, aph(3”)-Ib, QnrB19, sul2, tet(A), dfrA14*	Col(pVC)	S	ND	R	R	ND
			2015	*aph(6)-Id, aph(3”)-Ib, QnrB19, sul2, tet(A), dfrA14*	Col(pVC)	S	ND	R	R	ND
			2015	*aph(6)-Id, aph(3”)-Ib, QnrB19, sul2, tet(A), dfrA14*	Col(pVC)	S	ND	R	R	ND
			2016	*aph(6)-Id, aph(3”)-Ib, QnrS1, floR, sul2, tet(A), dfrA14*	IncN_ST7	S	R	R	R	R
			2016	*aph(6)-Id, aph(3”)-Ib, QnrS1, floR, sul2, tet(A), dfrA14*	IncN_ST7	S	R	R	R	R
			2016	*aph(6)-Id, aph(3”)-Ib, sul2, tet(A), dfrA14*	Col(pVC)	S	S	S	R	R
			2016	*aph(6)-Id, aph(3”)-Ib, sul2, tet(A), dfrA14*	Col(pVC)	S	ND	S	R	ND
			2016	*QnrB19*	Col(pVC)	S	S	R	S	S
			2016	*QnrB19*	Col(pVC)	S	S	R	S	S
		Senegal (n = 1)	2012	*aph(6)-Id, aph(3”)-Ib sul2, tet(A), dfrA14*	IncI1_ST21	S	S	S	R	R
Asia 1^a^ (n = 11)	ST343 (n = 11)	Thailand (n = 6)	2008	*bla_TEM-1B_, QnrS1, gyrA* p.S83F	IncN	R	S	R	S	S
			2008	None	None	S	S	S	S	S
			2009	None	None	S	S	S	S	S
			2010	*gyrA* p.S83F	None	S	S	R	S	S
			2012	None	None	S	S	I	S	S
			2013	*aadA1, sul3*	IncI1_ST3	S	S	S	S	S
		None (n = 5)	2003	*bla_TEM-1B_, QnrS1*	IncN	R	S	R	S	S
			2010	None	None	S	S	I	S	S
			2010	None	None	S	S	S	S	S
			2012	None	None	S	S	I	S	S
			2015	*dfrA14*	IncFII, Col(pVC)	S	S	I	S	S
Asia 2^a^ (n = 8)	ST2063 (n = 8)	Sri Lanka (n = 4)	2011	*bla_TEM-1B_*	IncL/M(pOXA-48)	R	S	I	S	S
			2012	*aph(6)-Id, bla_TEM-1B_, QnrS1, sul3, tet(A), dfrA14*	IncX1	R	S	R	R	R
			2012	None	None	S	S	I	S	S
			2016	*QnrB19*	Col(pVC)	S	ND	I	S	ND
		Thailand (n = 2)	2015	None	Col(pVC)	S	ND	I	S	ND
			2016	None	Col(pVC)	S	ND	S	S	ND
		Not known (n = 1)	2014	None	Col(pVC)	S	S	I	S	S
		None (n = 1)	2015	None	Col(pVC)	S	ND	I	S	ND

BL: β-lactam antibiotics; CHL: chloramphenicol; I: intermediate resistance; ND: not done; QNL: quinolone; R: resistant; S: susceptible; ST: sequence type; SXT: trimethoprim-sulfamethoxazole; TET: tetracycline; WGS: whole genome sequencing.

^a^ WGS clusters are named for the geographical regions where the majority of cases had travelled. Asia 1 and Asia 2 refer to two separate phylogenetic clusters we identified among the cases with travel history to Asia.

**Figure 2:**
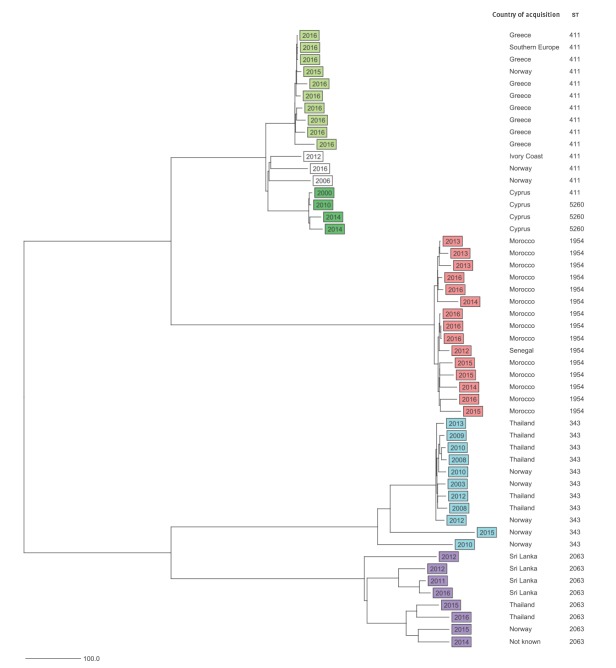
Neighbour-joining tree of *Salmonella* Chester isolates, based on 3,009 core genes included in core genome multilocus sequence typing Norway, 2000–2016 (n = 51)

All 51 *S*. Chester isolates had ≥ 98.6% good cgMLST targets (mean: 99.4%). Through cgMLST, based on 3,002 core genes and seven MLST genes, we identified four phylogenetic clusters separated by ≥ 719 allelic differences. These clusters were primarily associated with different geographical regions of acquisition: Europe, Africa and two separate clusters for Asia (Figure 2, Table). The European and Asian clusters included isolates from cases without reported history of travel outside Norway (Figure 2). Half of the cases in the Asia 1 cluster had a history of travel to Thailand (6/11) and in the Asia 2 cluster to Sri Lanka (4/8). The European cluster was further divided into Greek (n = 10) and Cypriot (n = 4) subclusters (Figure 2), while three isolates belonged to neither subcluster. The European subclusters were distanced from each other by ≥ 107 allelic differences. Within each of the two Asian clusters, the allelic differences between isolates were present in up to 8.2% (248/3,009) of the included genes, and within the European cluster in 4.2% (107/3,009) of the genes. There were fewer allelic differences between the isolates within the Greek (1.3%; 40/3,009) and Cypriot (0.9%; 27/3,009) subclusters and within the African cluster (1.5%; 45/3,009). Some identical isolates were also present: three isolates in the Greek subcluster were identical by cgMLST, as were two isolates in the African cluster. The years of isolation of *S.* Chester from the different clusters overlapped in time (Figure 2).

Nodes are labelled according to year of isolation. In addition, country of acquisition and seven-gene sequence type (ST) is noted. Scale bar shows absolute number of allelic differences. Green and white: European cluster (ST411 and ST5260); red: African cluster (ST1954); blue: Asian cluster 1 (ST343); lilac: Asian cluster 2 (ST2063). The analysis was performed in in Ridom SeqSphere^+^.

### Single nucleotide polymorphism-based reference mapping analysis

**Figure 3:**
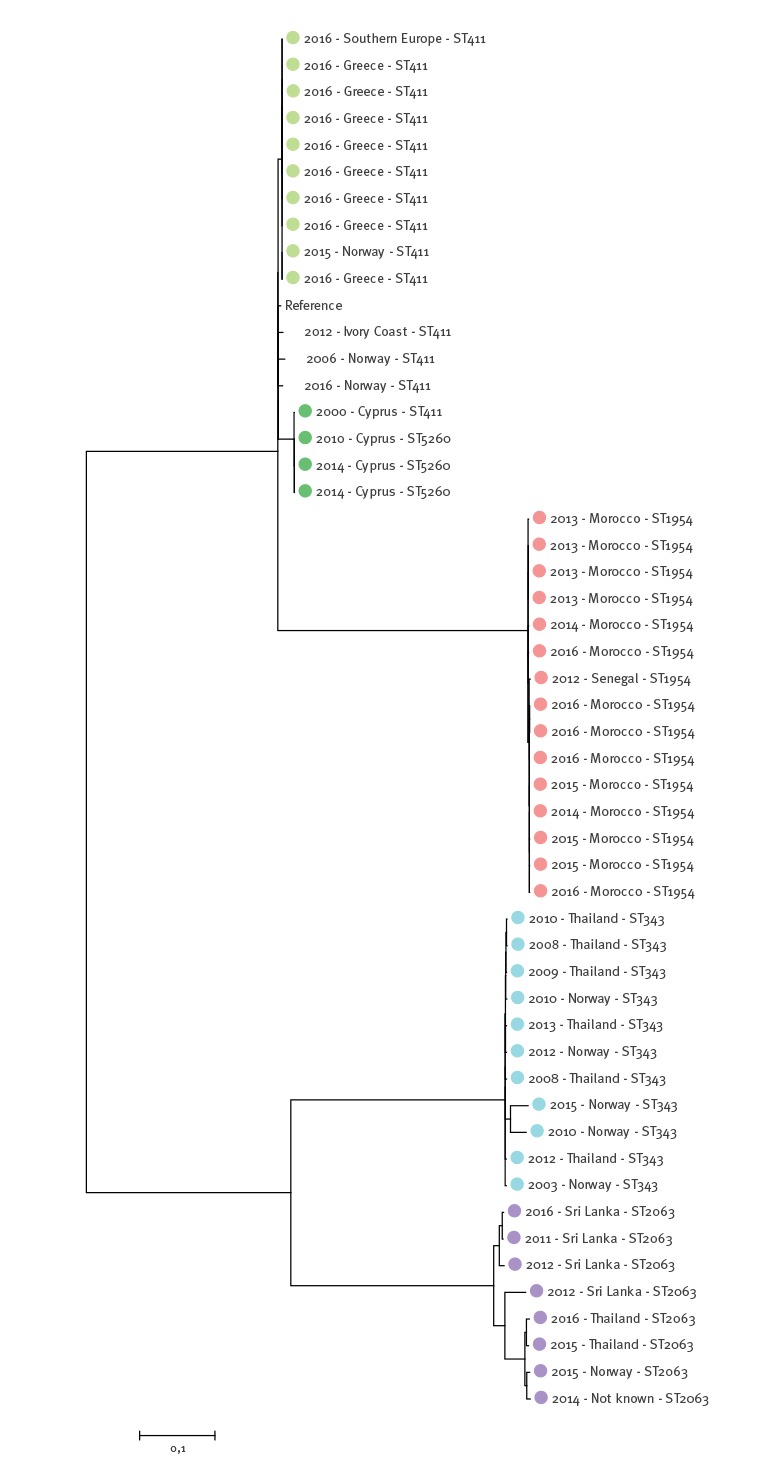
Molecular phylogenetic analysis of *Salmonella* Chester isolates, based on single nucleotide polymorphism differences, Norway, 2000–2016 (n = 51)

Genome mapping phylogeny based on 14,176 SNPs revealed four main clusters that corresponded with the cgMLST results (Figure 3). The clusters were separated by ≥ 3,623 SNPs. Within the European cluster as a whole, there were 416 SNP differences. While within the Greek and Cypriot subclusters, there were ≤ 8 and ≤ 16 SNP differences, respectively, these subclusters were separated from the other three isolates within the European cluster by ≥ 170 SNPs. Within the African cluster, there were ≤ 51 SNP differences, while there were ≤ 601 and ≤ 852 SNP differences, respectively, within the Asia 1 and Asia 2 clusters.

The branch structure was confirmed by a bootstrap consensus tree inferred from 1,000 replicates. A total of 14,176 positions were present in the final dataset. *S.* Chester SRX992125 was used as a reference. Nodes are labelled according to year of isolation, country of acquisition and seven-gene sequence type (ST). Green and white: European cluster (ST411 and ST5260); red: African cluster (ST1954); blue: Asian cluster 1 (ST343); lilac: Asian cluster 2 (ST2063). The scale bar shows substitutions per site.

### Discriminatory power

The discriminatory power by Simpson’s index of diversity was 0.78 for conventional MLST and 0.99 for cgMLST and genome mapping.

### Antimicrobial resistance by phenotypic and genotypic characterisation

Overall, 16 of the 51 isolates were quinolone resistant by phenotypic testing. All 16 carried one or more quinolone resistance determinants: 10 carried the *qnrS1* gene and five carried the *qnrB19* gene, and the S83F SNP in *gyrA* was identified in two isolates, one of which also carried *qnrS1* (Table). All isolates resistant to chloramphenicol (6/6), trimethoprim-sulfamethoxazole (14/14) and tetracycline (9/9) carried known resistant determinants to these antimicrobials. The four isolates in this study that were resistant to β-lactams carried *bla_TEM-1B_* and were present in the two Asian clusters. Antimicrobial resistance varied between the clusters identified through WGS (Table). No isolates in the European cluster were fully resistant to any of the tested antimicrobials. 

All isolates in the African cluster were resistant to at least one class of antibiotics. Most common in this cluster was trimethoprim-sulfamethoxazole resistance (n = 13), these resistant isolates carried both the *sul2* and *dfrA14* genes. Thirteen isolates in the African cluster were resistant to two or more classes of antibiotics. Twelve isolates in this cluster harboured the full set of resistance genes (*aph(3”)-Ib (strA)*, *aph(6)-Id (strB)*, *sul2*, *tet(A)*, and/or *floR*) carried on the Tn3-like transposon that was identified in the outbreak cluster associated with travel to Morocco in a previous study [2]. 

Resistance to multiple antimicrobial agents was rare outside the African cluster. However, in the Asia 1 cluster, two isolates displayed resistance to both β-lactams and quinolones and carried the *bla*_TEM-1B_ and *qnrS1* genes. In the Asia 2 cluster, one isolate carried the IncX1 plasmid and the *aph(6)-Id (strB)*, *bla_TEM-1B_*, *qnrS1*, *sul3*, *tet(A)*, *dfrA14* genes, and was resistant to β-lactams, tetracycline, quinolone and trimethoprim-sulfamethoxazole. Across all the clusters, colicin bacteriocin-encoding Col-plasmids were carried by 25 of the 51 isolates. Plasmids of the incompatibility (Inc) types detected among the isolates were IncI1, IncI2, IncX1, IncX3(pEC14), IncFII, IncL/M(pOXA-48) and IncN. These were carried by 16 of the 51 isolates (Table). In the African cluster, six of 15 isolates carried the IncN-pST7 plasmid, which was not found in any of the other clusters.

## Discussion

It is widely recognised that WGS-based methods offer higher resolution compared with conventional typing methods in distinguishing outbreak-associated isolates from sporadic ones [22,23]. For several enteropathogenic bacterial species, high concordance of results has been shown between cgMLST and reference mapping approaches, including *Salmonella* Enteritidis [24], *Listeria* [25] and *Enterococcus faecium* [26]. We observed similar concordance in our study, where the same four clusters were identified by both approaches. Both cgMLST- and SNP-based analyses identified the same Greek and Cypriot subclusters within the European cluster. The allelic and SNP differences observed in the two workflows also both confirmed the same phylogeny, where the European and African clusters were more similar to each other and more distant from the two Asian clusters. There was more internal diversity within the Asian clusters, compared with the internal diversity within the European subclusters and within the African cluster. The four main clusters identified through WGS displayed distinct STs based on conventional MLST for seven housekeeping genes, although the Cypriot subcluster within the European cluster included both ST411 and a novel SLV ST5260. 

The discriminatory power of cgMLST- and SNP-based analyses was high and exceeded that of conventional MLST. The discriminatory power of cgMLST, combined with the ease of performing the analysis, the lower requirements of computational power and bioinformatics knowledge compared with a reference mapping SNP-based workflow, makes this an appropriate method for public health microbiology. Isolates can be analysed and compared with previously analysed isolates as they are received, which allows for continuous monitoring of potential outbreak clusters through gene-by-gene comparisons of a standardised cgMLST. In addition, because the scheme is standardised, the cgMLST EnteroBase can be used to describe the analysed isolates in a wider context by comparing them to other analysed isolates. For further analysis of clusters detected by cgMLST, SNP analysis can be performed for even greater resolution.

In this study, we used cgMLST to investigate genetic relatedness of *S.* Chester for surveillance and early outbreak detection and to compare the isolates from Norwegian patients who had travelled to Greece with isolates from patients with a history of travel to other geographic regions. As the majority of the *Salmonella* cases identified in Norway report a history of travel [27], our data offer some level of insight into the internationally circulating *S.* Chester strains. 

The travel history of our cases allowed us to identify geographical clusters, and our results also show that unrelated clusters, describing probable outbreaks, were overlapping in time. For example, the isolates from the African cluster, identified in the period between 2012 and 2016, were unrelated to the isolates associated with travel to Rhodes, Greece, which were identified in the summer of 2016. The isolates from cases who had travelled to Greece formed a separate group within the larger European cluster. The European cluster also included a smaller Cypriot subcluster, with four isolates from 2000, 2010, and 2014. 

To further investigate the European cluster, we compared the ST411 isolates included in this study with the ST411 isolates deposited in EnteroBase. Of the 14 ST411 isolates included in our study, 10 clustered together with fewer than two allelic differences. They represent the Greek subcluster within the European ST411 cluster that we identified in our study, and the reported travel history of the 10 cases was Greece (n = 8), southern Europe (n = 1) and none (n = 1). In addition to our isolates, five ST411 isolates from the UK clustered within two allelic differences from our Greek subcluster, however, the travel history was unknown for the isolates from the UK.

While this and previous studies indicate that both SNP- and cgMLST-based WGS analysis can provide epidemiologically relevant microbiological information in the context of an outbreak investigation, it does not replace epidemiological information. In any outbreak investigation, microbiological and epidemiological data ideally complement each other in disentangling the outbreak, but microbiological data like these, especially when performed regularly as molecular surveillance, may alert to potential outbreaks that require epidemiological investigation. Although interviews were unable to confirm a source or common exposure for the cases travel-related to Rhodes, Greece, the WGS results of both the SNP-based and the cgMLST analysis give reason to believe that these cases constituted an outbreak. Surprisingly, our EPIS enquiry did not return any reports from similar findings elsewhere in Europe, although Rhodes is a holiday destination for many Europeans. 

The isolates within the African cluster and within the Cypriot subcluster were genetically similar, although spanning several years, and isolates such as these should be flagged in WGS-based molecular surveillance for possible further investigation. In contrast, the two Asian clusters revealed through WGS in our study were geographically less contained, with cases reporting travel to one of several Asian countries or no travel abroad, and spanned a period of several years. They were also genetically more diverse and probably do not represent outbreak clusters, but rather a sample of the strain population that circulates in Asia and perhaps elsewhere.

As WGS is increasingly employed in public health microbiology to provide epidemiologically relevant information for outbreak investigations and surveillance, harmonised or standardised cut-offs for cluster definitions are needed and have already been proposed for some species [28]. Because of the inherent characteristics of the WGS analysis methods, we can expect that the SNP variation will be greater than the allelic differences in the same cluster, therefore the cut-offs must be adjusted not only to the species under investigation and possibly to subtype, serovar or serotype, but also to the WGS analysis approach. In addition, SNP-based results may differ from each other depending on trimming and pruning quality parameters defined in the SNP identification process, and some suggest that it may be impossible to define single cut-off values for outbreaks [14,29]. For cgMLST, a cut-off value for clusters would most probably need to take into account the number of core genes included in the analysis, and perhaps the cut-off could be a percentage of allelic differences rather than an absolute number. However, even for cgMLST, different assembly software could introduce some variation into the results, even when using the same sequencing chemistry.

The clusters associated with travel to Europe and Asia included cases without travel history. However, domestically acquired salmonellosis cases are rare in Norway [27]. These results therefore invite speculation on the possibility of secondary transmission to people in Norway from persons with travel history or through consumption of imported food items. Previous studies on salmonellosis have concluded that most cases are contracted through contaminated food, while person-to-person transmission is rare [30]. Inadvertent omission of travel details in connection with specimen collection or isolate submission is also a possibility that could explain these results. 

In our study, two clusters were related to travel to Asia, one with just over half of the cases reporting travel to Thailand, and the other with travel history to Sri Lanka or Thailand. Geographical clusters were also identified in a previous study focusing on the multinational outbreak of *S.* Chester in Europe related to travel to Morocco in 2014 and 2015 [2], however, our study indicates that the outbreak may have been still ongoing in late 2016, as four cases belonging to the cluster and reporting travel to Morocco were identified in Norway in November 2016.

Antimicrobial resistance varied between the clusters. Aside from six intermediately quinolone-resistant isolates, all isolates in the European cluster were susceptible to all tested antibiotics, while some resistance was seen in the two Asian clusters. The three isolates displaying resistance to both quinolones and β-lactams and one isolate resistant to β-lactams were part of the two Asian clusters. The results for the isolates from the African cluster, where resistance to antibiotics was frequent, are in agreement with prior knowledge about the ST1954 cluster [2]. It has been concluded that the use of antibiotics in treating non-severe *Salmonella* diarrhoea offers no clinical benefits and that antibiotics appear to increase adverse effects and may prolong the presence of *Salmonella* [31]. However, as antibiotic use plays an important role in the development of antibiotic resistance, this variation between clusters may reflect variation in the use of antimicrobials for humans and livestock between the originating regions. Estimating global consumption of antimicrobials in animals is challenging, but experts estimate that it will increase by 67% from 2010 to 2030 [32]. Studies describing findings of *S.* Chester from animal feed and faeces are available for two African countries. In a study of *Salmonella* in animal feed commercially produced in Namibia, *S.* Chester was the most commonly encountered serovar; however, resistance was rare. In a separate study, *S.* Chester isolates with intermediate resistance to streptomycin were discovered in poultry and cattle faeces in Burkina Faso [33,34].

A previous study by Fonteneau et al., focusing on the multinational *S.* Chester outbreak related to Morocco, found that isolates carrying the IncN-*qnrS1* plasmid appeared in 2014 [2]. In our material, isolates harbouring this plasmid were isolated already in 2013. In our study, these isolates were also ST1954 and originated from cases with history of travel to Morocco, which indicates that one of the sources in the multisource outbreak may have been active already then. As IncN plasmids are more commonly identified in isolates from animals than from humans, it has previously been suggested that the plasmids could have acquired the *qnrS1* gene in animals [35]. Six of our isolates harboured the same IncN-pST7 plasmid that was first reported in isolates connected to the Moroccan outbreak [2]. Two thirds of the resistant isolates in our study carried plasmids that have been linked with plasmid-mediated quinolone resistance [35]. All fully quinolone resistant isolates carried one of the *qnr* genes and/or point mutations known to confer resistance. The Inc plasmid types identified in our study were not confined to one WGS cluster, and the isolates in a cluster did not all carry the same plasmids.

A limitation of our study is the convenience sample of *S.* Chester isolates included in the analysis. However, we have attempted to mitigate this by including all isolates submitted to the National Reference Laboratory in the years from 2014 to 2016, and the additional isolates were selected to represent multiple years and a variety of geographical origins. A second limitation of our interpretation of the results is that we do at this point not have universal defined cut-offs of the number of SNP or allelic differences to determine clusters for *S.* Chester. However, we believe that in the future, as WGS continues to be used and more genomes become available in the public databases, our possibilities to determine exact cut-offs for defining a cluster will improve through sharing data such as those we obtained in this study.

## Conclusion

WGS of *S*. Chester cases in Norway shows geographically distinct clusters associated with travel history of the patients and with varying antimicrobial susceptibility profiles between clusters. Although standardised cut-off values for relatedness as defined through WGS need more epidemiological validation and further data, our results indicate an outbreak of *S*. Chester in Norway during summer 2016. They further indicate that the outbreak was related to travel to Rhodes, Greece, and different from the simultaneous multicountry outbreak associated with travel to Morocco [2]. We recommend implementing cgMLST-based molecular surveillance for accurate and timely detection of future outbreaks for *S.* Chester and other *S. enterica* isolates.
